# Impact of Residential Mobility on Exposure Assessment in Longitudinal Air Pollution Studies: A Sensitivity Analysis within the ESCAPE Project

**DOI:** 10.1100/2012/125818

**Published:** 2012-11-28

**Authors:** Anna Oudin, Bertil Forsberg, Magnus Strömgren, Rob Beelen, Lars Modig

**Affiliations:** ^1^Occupational and Environmental Medicine, Department of Public Health and Clinical Medicine, Umeå University, 901 87 Umeå, Sweden; ^2^Department of Geography and Economic History, Umeå University, 901 87 Umeå, Sweden; ^3^Institute for Risk Assessment Sciences, Utrecht University, P.O. Box 80178, 3508 TD Utrecht, The Netherlands

## Abstract

Exposure misclassification in longitudinal studies of air pollution exposure and health effects can occur due to residential mobility in a study population over followup. The aim of this study was to investigate to what extent residential mobility during followup can be expected to cause exposure misclassification in such studies, where exposure at the baseline address is used as the main exposure assessment. The addresses for each participant in a large population-based study (*N* > 25,000) were obtained via national registers. We used a Land Use Regression model to estimate the NO_*x*_ concentration for each participant's all addresses during the entire follow-up period (in average 14.6 years) and calculated an average concentration during followup. The Land Use Regression model explained 83% of the variation in measured levels. In summary, the NO_*x*_ concentration at the inclusion address was similar to the average concentration over followup with a correlation coefficient of 0.80, indicating that air pollution concentration at study inclusion address could be used as indicator of average air pollution concentrations over followup. The differences between an individual's inclusion and average follow-up mean concentration were small and seemed to be nondifferential with respect to a large range of factors and disease statuses, implying that bias due to residential mobility was small.

## 1. Introduction

Exposure assessment is crucial in all studies of environmental exposures. Exposure assessment error is typically denoted as exposure misclassification and may lead to bias both towards and away from the null. Exposure misclassification is especially important to consider in longitudinal studies, where typically, large populations are followed over many years, making exposure assessment complicated. In such studies, air pollution concentration at the home address of the study participant is often used as a marker for personal exposure to air pollution, even though there evidently are other factors and sources influencing the actual exposure [1, 2], such as subjects not always being at home and using outdoor concentrations without taking indoor concentrations into account.

Moreover, exposure misclassification can occur due to residential changes among a study population over followup (residential mobility). In longitudinal studies, the concentration at the address at study inclusion [3], the address at followup [4], or a combination of both [5] is typically used as a marker of average air pollution concentration over followup although it may be unreasonable to use the exposure at the home address at one or a few points in time as a constant measure of exposure [6]. Despite that, exposure based on each participant's full residential history is seldom assessed in air pollution epidemiology. In a recent longitudinal study on air pollution exposure, the effect estimates were stronger when accounting for residential mobility than when not [7]. Moreover, Gan and colleagues observed that reducing exposure by changing home address during a follow-up period reduces the risk of coronary heart disease in comparison to those with a more constant exposure throughout the entire followup [6].

Bias caused by residential mobility is related to length of followup, to how often study participants change home address, to spatial contrasts in exposure concentrations within the study area, and to the dependency between residential mobility and the outcome of interest. Moreover, impact of residential mobility on exposure misclassification can be expected to depend on age and geography and therefore needs to be further studied and described within different cohorts.

The aim of this study was to investigate to what extent residential mobility during followup can be expected to cause exposure misclassification in longitudinal studies of air pollution exposure using exposure at the baseline address as the main exposure assessment. The aim was also to investigate potential bias on estimated health effects caused by residential mobility.

## 2. Method

### 2.1. Population and Exposure Assessment

**Figure 1:**
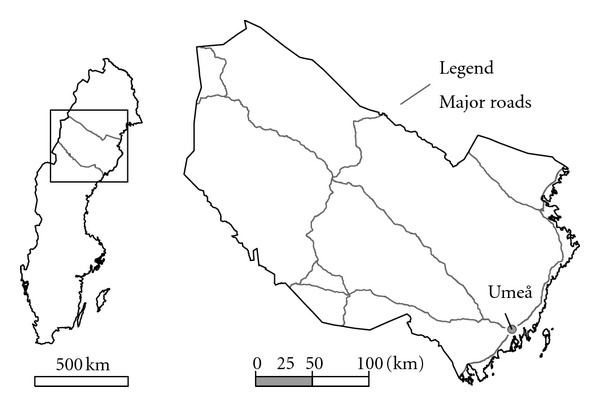
The Swedish county of Västerbotten.

Västerbotten county is participating in the EU-financed ESCAPE project (http://www.escapeproject.eu/) with the local part of the “European Prospective Investigation into Cancer and Nutrition” (EPIC) cohort, which consists of approximately 26,000 participants. The study population is recruited from the whole of the county, which comprises a large geographical area (ca 55,000 km^2^; about 260,000 inhabitants) in the northern part of Sweden (Figure 1). The largest city in the area is Umeå (approximately 80,000 inhabitants), which is situated at the coast in the southeastern part of the county.

The cohort was based on a random sample of clinically investigated men and women. The inclusion year ranged from 1992 to 1996. Thereafter, the participants were followed every year through medical registries and invited every 10th year for a new clinical investigation. The initial participation rate was 56.5%.

The participants' full residential histories during followup were collected from the Swedish nationwide population registry, using each participant's unique personal identification number. From the registry, each participant's address at the end of each year during followup was collected and geocoded, meaning that several residential changes done within a calendar year could thus not be captured.

Within the ESCAPE project, a Land Use Regression model (LUR) was developed for all participating cities according to a predefined procedure developed by the project (http://www.escapeproject.eu/manuals/ESCAPE_Exposure-manualv9.pdf). The LUR model covered the whole Västerbotten county, including the main city of Umeå. Details concerning the model procedure are described in forthcoming publications [8, 9]. Shortly, the model uses geographical information to explain the geographical variation of nitrogen oxides (NO_*x*_) and nitrogen dioxides (NO_2_), both markers of traffic-related air pollution. The model was developed based on measurements of NO_2_ and NO_*x*_ made at measuring sites representative for the geographical area of interest. The models explained 87% of the variability. “Leave one out” cross-validation was used for validating the models, where the NO_2_ model explained 83% of the variation and the NO_*x*_ model 82% of the variation in measured levels. The model can be used to estimate the levels of NO_2 _ and NO_*x*_ in any given geographical coordinate within the area.

To be able to investigate the impact of residential mobility, we used the LUR model to estimate the concentration for each participant's all addresses during the entire follow-up period and calculated an average concentration during followup. No adjustments were made to account for temporal change in exposure levels; thus all geographical coordinates were ascribed the exposure levels representative for the years of model development (2009-2010).

### 2.2. Statistical Methods

For each participant, the pollutant concentrations at the inclusion address and the average concentration during followup were calculated. Accordance between concentration at the inclusion home address and average concentration during followup was compared by calculating Pearson correlation coefficients and by producing scatterplots and histograms over the differences between the two measures. The two measures were also categorized into three categories, and the accordance between the categorized variables was expressed as kappa-values. Several different categorizations were investigated. A subanalysis restricted to the population of the main city Umeå (*N* = 8, 402), where the largest exposure contrasts were present, was done in order to investigate if accordance between the two exposure measures was influenced by living in a city or in a more rural area. Another subanalysis was done, restricted to study participants who changed home address at least once during followup. In order to investigate whether concentration differences between study inclusion and average over followup were differential with respect to common diseases, risk factors, and socioeconomy, we stratified data on the following variables: sex, age, body mass index categories, education level, hypertension, diabetes, cardiac disease, self-estimated health, smoking (all at study inclusion), and cancer during followup.

## 3. Results

**Figure 2:**
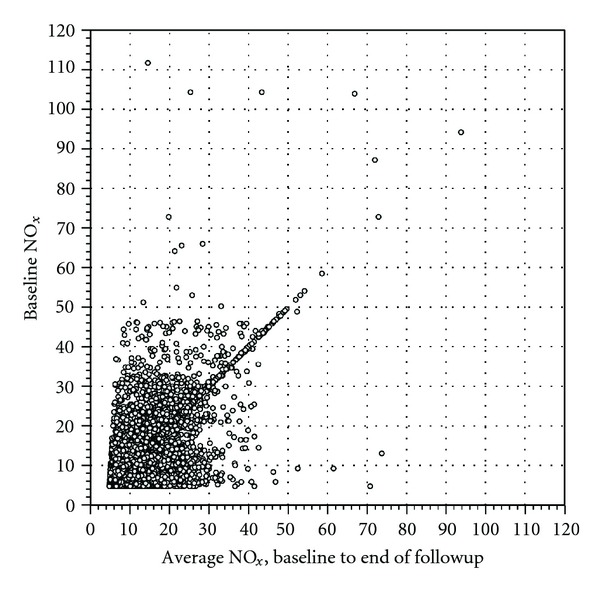
Scatterplot of the NO_*x*_ concentrations at inclusion versus follow-up average concentrations (Pearson correlation coefficient = 0.80).

**Figure 3:**
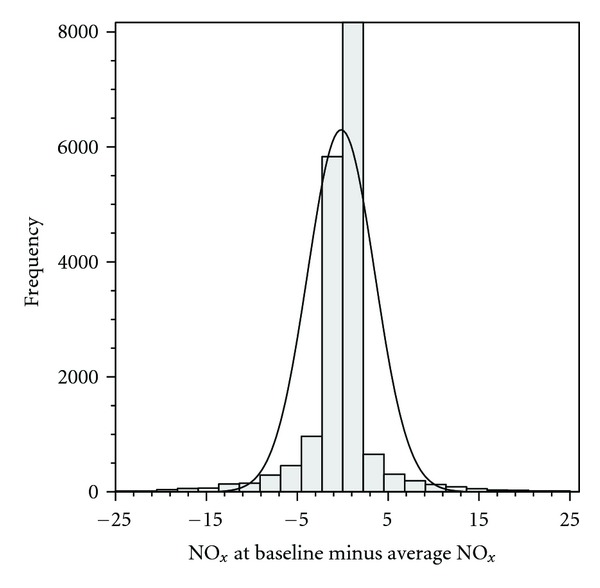
Histogram of NO_*x*_ concentration differences between inclusion and follow-up average concentrations. The highest bar represents those with a 0 or very small difference (*n* = 15, 962) and has been truncated.

The average length of followup was 14.6 years, and the average study participant had changed home address 1.75 times (Table 1). The average NO_*x*_ concentration from inclusion to followup seemed to be in good accordance with the concentrations at inclusion, with a correlation coefficient of 0.80 and with the scatterplot and histogram revealing generally small absolute differences between the two measures (Table 2 and Figures 2 and 3). Restricting the analysis to those who had changed home address at least once only marginally lowered the correlation (*ρ* = 0.76; Table 2). Moreover, the weighted kappa of 0.76 reveals substantial agreement for the categorized variables (Table 3). Other choices of categorization yielded similar kappa-values (data not shown). Not only were the absolute differences in concentration low, the relative differences were also rather small; more than 85% of the cohort had an average concentration over followup that differed less than 25% from the concentration at baseline. The NO_*x*_ concentrations over followup thus differed from the NO_*x*_ concentrations at baseline with more than 25% for 15% of the cohort, but over- and underestimation of the concentrations seemed equally frequent (Figures 2 and 3). The large number of study participants (approximately 16000) for whom the concentration at inclusion is identical to the average concentration during followup is explained by the fact that no temporal adjustment (back-extrapolation) of the concentrations has been undertaken. Thus, if a study participant had not changed home address during followup, the study inclusion concentration is per se equal to the average concentration.

A large part of Västerbotten county is rural with small exposure contrasts, but the analyses where data were restricted to the participants residing in the city Umeå revealed that the correlation coefficient was only marginally lower in Umeå than for the entire cohort (Table 2).

In the entire cohort, 17,896 out of 25,725 persons (70%) had changed residential address during followup. In Umeå, the corresponding numbers were 7,020 out of 8,402 (84%). Analysis restricted to study participants who changed home address at least once did not reveal any substantial changes in correlation (*ρ*_entire cohort_ = 0.76, *ρ*_Umeå_ = 0.74).

The relative differences were slightly higher in Umeå than in the entire cohort; 77% compared to 85% of the population had an average concentration over followup that differed less than 15% from concentration at baseline. When restricting the population to those who has changed residential address during followup, the proportion was 78% in the entire cohort and 73% in the Umeå cohort.

The results regarding NO_2_ were overall very similar to the NO_*x*_ results (data not shown).

The difference in NO_*x*_ concentrations between average and study inclusion concentration did not depend on factors such as sex, age, body mass index, education level, hypertension, diabetes, cardiac disease, self-estimated health, smoking, or cancer during followup, implying that exposure misclassification caused by residential mobility is nondifferential (data not shown).

## 4. Discussion

Exposure misclassification to some extent is inevitable in longitudinal studies on health effects of air pollution exposure, since it is not feasible to conduct large population-based studied spanning over many years in time where each person carries a personal measuring device. Moreover, the potential bias caused by exposure misclassification is difficult to assess. In a large population-based study based on register-data and LUR-modeled concentrations of air pollution, we observed that the concentrations of air pollution at the study subject's home addresses at study inclusion were similar to the average concentrations over the entire followup. The small differences observed between study inclusion and average follow-up concentration did not seem to be differential on the factors sex, age, body mass index, education level, hypertension, diabetes, cardiac disease, self-estimated health, smoking, or cancer during followup, indicating a nondifferential misclassification with respect to many common risk factors and diseases. In summary, the results, namely, that over- and underestimation of concentrations were equally frequent and rather small in amplitude and that misclassification due to residential mobility did not seem to depend on common risk factors and disease, imply that residential mobility did not seem to cause bias due to exposure misclassification in the present study setting. The results may be study area specific, but subanalyses, where the results did not differ substantially depending on urban/rural area or a wide range of other factors, seem to imply that, at least in the present study area, the results are rather homogeneous.

Exposure misclassification is a well-known source of bias in longitudinal epidemiological studies of air pollution health effects and has great importance for the accuracy of the results. The present study addresses one component of exposure misclassification, namely, residential mobility. The procedure of identifying the full residential history for a large cohort followed for long-time periods can be both time consuming and expensive. These results increase knowledge about the impact of residential mobility on exposure misclassification.

The study has several strengths and limitations that should be mentioned. A major strength is that the study is register based; data on home addresses were obtained from a national register which is considered to have very high data quality. The absolute majority of Swedes are present in the national registers, and the data are thus nearly complete. However, even though data quality overall is high, a certain proportion of the population is likely not residing at their official address. The exact proportion is difficult to estimate, but it is likely rather small and should thus have little impact on the results. The long follow-up time, in average 14.6 years, in combination with a large study population with more than 25,000 study subjects, is another strength of the present study. A potential weakness of the study is that, in contrast to what is assumed in the analysis, the concentration of air pollution has not been constant over time; a strong decline in most air pollutants has in fact been present throughout the study period. Most epidemiological studies using LUR models do not take into account such time trends, which makes this comparison realistic and the results important for these epidemiological studies. However, if large spatial variation in time trends were present, the validity of the results would decrease. Unpublished comparisons made locally in Umeå and comparisons in the Netherlands [10] have shown that, although the general levels of air pollution have been reduced over time, the spatial patterns have remained relatively similar. In Umeå this is explained by few large infrastructural changes with impact on the spatial distribution of traffic-related air pollution. Time trends in exposure should thus not affect the implication of the results of the present study.

The present study was based on parts of a large population-based study, where the whole population of the Västerbotten county was invited to participate. The participation rate was 56.5%. How generalizable are the results to other populations and to the persons who did not participate? Participation rates in general are known to depend on socioeconomic status, also in Sweden [11, 12]. The results of the present study could thus not be immediately generalized to the proportion of the population that did not participate. It is likely that residential mobility depends on factors such as socioeconomic position, nationality, and age, to mention a few. Air pollution concentrations are associated with socioeconomy, but size and direction of the association seem to differ between populations [2, 13]. It is therefore possible that changes of home address during followup would cause differential exposure misclassifications in other populations. However, in the present study, the relation between follow-up average concentration and inclusion concentration did not seem to depend on factors related to socioeconomy or disease status, indicating that the results were stable across different segments of the study population. Although people changed home address, they generally did not seem to change address to areas where concentrations were much different from their original address. However, we would encourage similar studies in other populations, although we are aware that they can be difficult to undertake in countries without national registers on the citizens' home addresses.

In summary, the NO_*x*_ and NO_2_ concentrations at the study inclusion address were similar to the average concentration over followup in this large register-based study of a northern Sweden population, indicating that air pollution concentration at study inclusion address could be used as indicator of average air pollution concentrations over followup. The differences between study inclusion and average follow-up concentrations were small and seemed to be nondifferential with respect to a large range of factors and disease statuses, implying that bias due to residential mobility was small.

## Figures and Tables

**Table 1:** Descriptive data of the cohort (25,725 observations).

	*N*	(%)
Inclusion year		
1992	2,139	8
1993	6,356	25
1994	5,793	23
1995	6,157	24
1996	5,280	21
Sex (men)	12,431	48

	Mean (SD)	5th to 95th percentile

Age at inclusion	46.0 (10.4)	30–60
Follow-up time	14.6 (3.6)	5–18
Number of changes of address from study inclusion to 2010	1.75 (2.34)	0–7

**Table 2:** Pearson correlation coefficients between study inclusion and follow-up average NO_*x*_ concentration at home address (*ρ*).

All study participants	Study participants who changed home address at least once	All study participants residing in the major city, Umeå	Study participants in Umeå who changed home address at least once
0.80	0.76	0.76	0.74

**Table 3:** Cross-tabulation between NO_*x*_ categorized into <75th percentile, 75th to 90th percentile, and ≥90th percentile (*μ*g/m^3^).

		NO_*x*_ in average over followup		
		<11.4	11.4–16.14	≥16.15
NO_*x*_ at inclusion	<9.7	17,870	929	440
	9.7–15.0	548	2,894	356
	≥15.1	186	430	1,947

The kappa-value was 0.73, and the weighted kappa-value was 0.76.
